# Comparative *in vitro* evaluation of native Indonesian macroalgae on rumen fermentation characteristics, digestibility, gas production kinetics, and enteric methane mitigation in ruminants

**DOI:** 10.14202/vetworld.2026.693-713

**Published:** 2026-02-23

**Authors:** Dimar Sari Wahyuni, Komang Gede Wiryawan, Roni Ridwan, Gunawan Gunawan, Arnold Parlindungan Sinurat, Maman Surachman, Rusli Fidriyanto, Ainisya Fitri, Dicky Pamungkas, Wisri Puastuti, Galih Kusuma Aji, Yeni Widiawati, Anuraga Jayanegara

**Affiliations:** 1 Study Program of Nutrition and Feed Science, Graduate School of IPB University, Bogor 16680, Indonesia; 2 Research Center for Animal Husbandry, National Research and Innovation Agency (BRIN), Bogor, Indonesia; 3 Department of Animal Nutrition and Feed Technology, Faculty of Animal Science, IPB University, Bogor 16680, Indonesia; 4 Research Center for Applied Zoology, National Research and Innovation Agency (BRIN), Bogor, Indonesia; 5 Research Center for Process Technology, National Research and Innovation Agency (BRIN), Bogor, Indonesia

**Keywords:** comparative macroalgae, enteric methane mitigation, gas production kinetics, *in vitro* rumen fermentation, macroalgae digestibility, ruminant nutrition, volatile fatty acids, sustainable livestock production

## Abstract

**Background and Aim:**

Enteric methane emissions from ruminants contribute significantly to greenhouse gas production, prompting research into sustainable feed additives. Macroalgae, rich in bioactive compounds, show promise in modulating rumen fermentation, improving digestibility, and reducing methane output. Indonesia's diverse native macroalgae remain largely unexplored for these purposes, necessitating comparative evaluation to identify promising species for ruminant nutrition. This study aimed to conduct the first comparative in vitro evaluation of rumen fermentation patterns, digestibility characteristics, gas production kinetics, and enteric methane emissions using 14 native Indonesian macroalgae species, including brown (*Sargassum sp., Padina sp., Turbinaria ornata*), green (*Boergesenia forbesii, Caulerpa racemosa, Ulva lactuca*), and red (*Palmaria palmata, Gelidium sp., Halymenia durvillei, Gracilaria verrucosa, Eucheuma cottonii, Gracilaria gigas, Eucheuma spinosum, Gracilaria coronopifolia*) algae, to identify candidates for sustainable ruminant feed additives.

**Materials and Methods:**

Macroalgae samples were collected from various Indonesian locations, dried, and analyzed for chemical composition (dry matter, ash, crude protein, crude fat, crude fiber, nitrogen-free extract). *In vitro* fermentation was performed using a completely randomized design with five replicates per species. Samples (0.5 g) were incubated at 39°C for 72 h in buffered rumen fluid from fistulated Ongole crossbreed cattle. Parameters measured included total gas production, methane emissions (estimated via volatile fatty acid [VFA] profiles), ammonia, total and partial VFAs (acetate, propionate, butyrate, valerate, iso-butyrate, iso-valerate), acetate-to-propionate ratio, *in vitro* dry matter digestibility (IVDMD), *in vitro* organic matter digestibility (IVOMD), partitioning factor, microbial protein synthesis, and gas production kinetics. Data were analyzed using a one way analysis of variance with significance at p < 0.05 or p < 0.01, followed by post-hoc tests.

**Results:**

Chemical composition varied widely; red algae like Palmaria palmata had high crude protein (22.39 % dry matter), while brown algae like Padina sp. were ash-rich (74.39 % dry matter). Total gas production was highest in B. forbesii (54.75 mL; p < 0.01) and lowest in T. ornata (10.94 mL). Methane emissions and methane per incubated dry matter were lowest in Sargassum sp. (1.87 mM and 3.75 mM/g dry matter; p < 0.01), with Sargassum sp. and C. racemosa reducing methane by 71.86 %. Ammonia levels were similar across species (p > 0.05). Total VFA and propionate were highest in H. durvillei and B. forbesii (p < 0.01), with reduced acetate-to-propionate ratios. IVDMD and IVOMD were highest in H. durvillei (81.72 % and 69.53 %; p < 0.01). Gas kinetics showed B. forbesii with the highest asymptote (201.97 mL; p < 0.01) but slowest rate (0.01 mL/h). Positive correlations existed between crude protein and VFA/ammonia, while crude fiber inversely correlated with gas production and digestibility.

**Conclusion:**

*H. durvillei* emerged as optimal for enhancing rumen fermentation and digestibility, while *Sargassum* sp. excelled in methane mitigation. These species hold promise as natural additives for reducing environmental impacts in ruminant production, warranting *in vivo* validation for optimal inclusion rates and long-term effects.

## INTRODUCTION

The increasing global demand for sustainable livestock production has intensified research into alternative feed resources capable of mitigating the environmental footprint of ruminant agriculture, particularly by reducing enteric methane (CH_4_) emissions. Macroalgae have attracted considerable attention as a promising dietary intervention due to their distinctive biochemical profiles, capacity to modulate rumen fermentation patterns, and documented potential to suppress methanogenesis [1]. Macroalgae exhibit remarkable morphological and functional diversity and are taxonomically classified into three major groups: brown algae (Phaeophyceae, Heterokontophyta), red algae (Rhodophyta), and green algae (Chlorophyta) [2]. Their nutritional composition, including protein, minerals, polysaccharides, lipids, and bioactive secondary metabolites, varies substantially depending on species, geographic origin, season of harvest, and prevailing environmental factors such as light intensity, water temperature, and nutrient availability [3]. When included in ruminant diets, macroalgae can provide essential macro- and micronutrients while simultaneously influencing rumen microbial ecology and fermentation dynamics, often leading to improvements in animal health, nutrient utilization efficiency, and overall productivity [4]. These beneficial effects are primarily attributed to the presence of secondary metabolites—such as phlorotannins, polyphenols, and halogenated compounds (notably bromoform)—which directly inhibit methanogenic archaea, together with shifts in volatile fatty acid (VFA) profiles that enhance propionate production and improve hydrogen sink efficiency [5–7].

More recent investigations have revealed that macroalgae not only lower enteric methane output but also induce significant changes in rumen microbial community structure and gene expression. For example, red seaweeds belonging to the genus *Asparagopsis* (particularly *Asparagopsis armata* and *Asparagopsis taxiformis*) have been shown to downregulate key methanogenesis pathway genes and alter carbon catabolic profiles within the rumen microbiome [8]. Similarly, species of the brown algal genus *Sargassum* sp. appear to selectively promote propionate-producing bacteria while reducing populations associated with acetate formation [9]. Indonesia, with its exceptionally rich and biodiverse marine macroalgal flora, possesses numerous native species that remain largely uncharacterized in terms of their ruminant feed potential. Leveraging these locally abundant resources could offer a regionally relevant, cost-effective strategy to simultaneously improve rumen fermentation efficiency, enhance nutrient digestibility, and decrease greenhouse gas emissions from tropical ruminant production systems.

Despite the growing global interest in macroalgae as anti-methanogenic feed additives, several critical knowledge gaps persist, particularly in tropical and biodiverse regions such as Indonesia. Most published studies have focused on a limited number of cosmopolitan or commercially cultivated species (especially *Asparagopsis* spp. and certain *Ulva* and *Gracilaria* species), while the vast majority of native Indonesian macroalgae, many of which are abundant, locally harvested, and potentially rich in unique bioactive compounds, have received little to no attention in ruminant nutrition research. There is a notable scarcity of comparative data on the *in vitro* rumen fermentation characteristics, nutrient digestibility, gas production kinetics, and methane mitigation potential of multiple co-occurring native species from the same geographic region. Furthermore, the roles of these macroalgae as multifunctional feed ingredients (beyond methane reduction), such as sources of high-quality protein, immunomodulatory compounds, natural antioxidants, or mineral supplements, remain poorly explored. This lack of systematic, comparative evaluation hinders the identification of the most promising species and optimal inclusion strategies tailored to tropical ruminant production systems, where seasonal forage quality is often limiting and environmental sustainability is increasingly prioritized.

The primary aim of this study was to address the identified research gap by conducting the first comprehensive comparative *in vitro* evaluation of rumen fermentation parameters, nutrient digestibility, gas production kinetics, and enteric methane production across 14 native Indonesian macroalgae species representing brown, green, and red algal groups. Specifically, the experiment sought to: (i) characterize differences in total and cumulative gas production, fermentation rate constants, and kinetic parameters; (ii) quantify variation in total and individual volatile fatty acid profiles, ammonia concentration, and acetate:propionate ratios; (iii) determine *in vitro* dry matter and organic matter digestibility; (iv) estimate methane emissions and identify species with the strongest anti-methanogenic activity; and (v) identify the most promising candidate(s) for future inclusion as natural feed additives capable of improving rumen function while simultaneously mitigating enteric methane emissions in ruminant production systems.

## MATERIALS AND METHODS

### Ethical approval

Rumen fluid containing solid particles was used as the source of microbial inoculum in this study. Rumen fluid was collected from three Ongole crossbreed cattle (*Peranakan Ongole*) maintained at the National Research and Innovation Agency (BRIN), Cibinong, Indonesia. The cattle were three years old with an average body weight of approximately 300 kg. Animals had ad libitum access to water and were fed three times daily, with dry matter consumption equivalent to 2%–3% of body weight and a roughage-to-concentrate ratio of 50%:50%. All animal handling and rumen fluid collection procedures complied with national animal welfare regulations and were approved by the Animal Ethics Committee of the National Research and Innovation Agency (approval no. BRIN-011/KE.02/SK/06/2022).

### Study duration and location

The *in vitro* experiment was conducted from July 2022 to February 2023 at the Laboratory of Annex and the Laboratory of Genomics, National Research and Innovation Agency (BRIN), Cibinong, Bogor, West Java, Indonesia. Additional analytical procedures were carried out at the Laboratory for Agro-Industrial and Biomedical Engineering Development, Serpong, South Tangerang, Indonesia.

### Collection and identification of macroalgae

**Figure 1:**
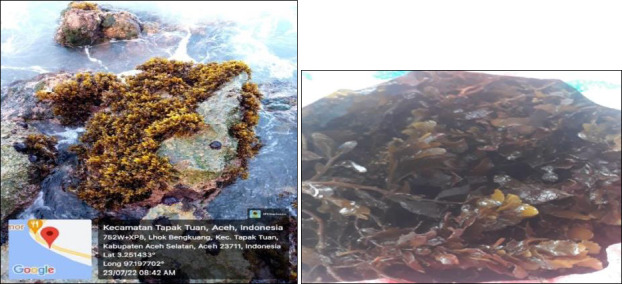
Sampling locations of *Sargassum* sp. and other macroalgae species used in the study.

A total of 14 macroalgae species were evaluated, namely *Sargassum* sp., *Palmaria palmata*, *Gelidium* sp., *Padina* sp., *Halymenia durvillei*, *Boergesenia forbesii*, *Gracilaria verrucosa*, *Eucheuma cottonii*, *Gracilaria gigas*, *Eucheuma spinosum*, *Ulva lactuca*, *Turbinaria ornata*, *Caulerpa racemosa*, and *Gracilaria coronopifolia*. Macroalgae were collected from South Aceh (Tapak Tuan), Makassar (Takalar), West Java (Sukabumi and Kronjo, Tangerang), East Java (Sidoarjo), Central Java, and Maluku (Larat). Additional material was purchased from CV. Winner Perkasa Indonesia Unggul during July–August 2022. Sampling locations are presented in Table 1. The sampling site and morphological characteristics of *Sargassum* sp. are shown in Figure 1.

**Table 1:** Some of the marine macroalgae species used in this study with sampling site location coordinates.

No.	Class	Order	Species	GPS coordinates	Sampling location
1	Florideophyceae	Halymeniales	*Halymenia durvillei* Bory de Saint-Vincent	7.1830° S, 131.7375° E	Maluku (Larat), Indonesia
2	Florideophyceae	Gracilariales	*Gracilaria gigas* Harvey	6°01’46” S, 106°26’30” E	Tangerang (Banten), Indonesia
3	Ulvophyceae	Bryopsidales	*Caulerpa racemosa* (Forsskål) Agardh	5.5966° S, 119.4832° E	Makassar (Takalar), Indonesia
4	Florideophyceae	Palmariales	*Palmaria palmata* (Linnaeus) F. Weber & D. Mohr	7.1830° S, 131.7375° E	Maluku (Larat), Indonesia

### Sample processing and storage

Macroalgae samples were subjected to a two-stage drying process consisting of sun drying for three days followed by oven drying at 55°C for four days. Dried samples were ground using a 0.5-mm disc mill [10]. Sample preparation followed previously described drying and processing procedures [11]. The processed seaweed flour was used for chemical composition analysis and *in vitro* incubation. All samples were stored in transparent plastic containers at −20°C in the Laboratory for Development of Industrial Agro and Biomedical Technology, BRIN.

Taxonomic and phycological identification was conducted for selected macroalgae species, including *G. gigas*, *H. durvillei*, *C. racemosa*, and *P. palmata*, by a phycologist at the Oceanography Laboratory, National Research and Innovation Agency. Identification of the remaining species was based on published literature sources. Technical support was provided by E-Layanan Sains, BRIN. Voucher numbers issued for phycological identification were 103722, 103774, 104577, and 1044576.

### Chemical composition analysis of macroalgae

Dry matter, organic matter, ash, crude protein, crude fiber, and ether extract contents were determined using AOAC standard methods [12]. Proximate analysis was conducted in duplicate (n = 2) for each parameter. The *in vitro* analytical procedures followed established methodologies described previously [13]. The chemical composition of the macroalgae species is presented in Table 2.

**Table 2:** Chemical composition (% dry matter) of several macroalgal species used in the study.

Macroalgae species	% Dry matter	% Moisture	% Ash	% Crude protein	% Crude fat	% Crude fiber	% Nitrogen-free extract (NFE)
Brown seaweeds							
*Padina* sp.	96.88	3.22	74.39	4.46	0.54	8.71	11.91
*Sargassum* sp.	90.22	10.85	23.50	11.91	1.31	5.34	57.94
*Turbinaria ornata*	93.11	7.40	36.82	10.55	1.46	9.21	41.96
Green seaweeds							
*Boergesenia forbesii*	94.54	5.77	43.01	5.93	1.54	25.39	24.13
*Caulerpa racemosa*	92.54	8.07	43.23	11.36	1.31	5.21	38.90
*Ulva lactuca*	87.61	14.14	25.21	14.73	0.64	8.46	50.96
Red seaweeds							
*Eucheuma cottonii*	90.41	10.60	35.24	10.59	2.10	8.14	43.93
*Eucheuma spinosum*	90.08	11.01	30.84	5.19	0.84	6.14	56.99
*Gelidium* sp.	91.76	8.98	31.50	11.15	0.50	47.34	9.51
*Gracilaria coronopifolia*	91.65	9.11	20.60	7.18	0.71	15.89	55.61
*Gracilaria gigas*	89.48	11.76	11.97	13.01	0.26	8.12	66.64
*Gracilaria verrucosa*	91.50	9.28	14.08	11.44	1.95	9.51	63.02
*Halymenia durvillei*	83.01	20.47	59.47	10.77	0.66	3.31	25.79
*Palmaria palmata*	94.20	6.15	15.19	22.39	0.33	8.39	53.70

NFE was calculated as 100 - (% ash + % crude fiber + % crude fat + % crude protein). All values are expressed on a dry matter basis.

### *In vitro* incubation procedure

*In vitro* rumen fermentation was conducted to evaluate digestibility using a modified gas production technique [13]. Rumen fluid donors were three male fistulated Ongole crossbreed cattle. Rumen fluid was collected through the fistula prior to morning feeding at approximately 07:00 h. The collected rumen fluid was filtered through four layers of cheesecloth and maintained at 39°C in a thermos flask during transport to the laboratory. The microbial inoculum was processed within 15 min of collection.

McDougall buffer solution was preheated to 39°C and flushed with CO_2_ before mixing with rumen fluid at a ratio of 1:2 (v/v). For each incubation unit, 500 mg of substrate was placed into a 100-mL serum bottle, followed by the addition of 50 mL of buffered rumen fluid. Anaerobic conditions were established by CO_2_ flushing before sealing the bottles with rubber stoppers and aluminium crimps. Incubations were performed in a water bath at 39°C for 48 or 72 h. After 48 h, samples were centrifuged at 378 × *g* for 10 min to separate supernatant and solid residues.

### Gas production measurement and methane estimation

Cumulative gas production was recorded at 2, 4, 6, 8, 10, 12, 24, 48, and 72 h of incubation using calibrated 50-mL medical syringes (Thermo Fisher Scientific, USA). Bottles were manually shaken after each measurement. Blank bottles containing rumen fluid and buffer without substrate were included for baseline gas correction.

Methane production (CH_4_ mM) was estimated from VFA profiles using a stoichiometric equation described previously [14]:

CH_4_ = 0.5C_2_ + 0.5C_4_ − 0.25C_3_ − 0.25C_5_,

where C_2_, C_3_, C_4_, and C_5_ represent acetate, propionate, butyrate, and valerate, respectively. Estimated methane production per incubated dry matter (mM/g DM) was calculated by dividing total CH_4_ by the dry matter weight of the incubated substrate.

### Fermentation parameters and *in vitro* digestibility analysis

Rumen pH was measured immediately after incubation using a calibrated pH meter (BP3001, Trans Instruments). Supernatant and solid fractions were separated after incubation. Supernatants were analyzed for total VFA, SCFA profiles, acetate-to-propionate ratio, and NH_3_–N concentration, while solid residues were used to determine DMD and OMD.

Total VFA and SCFA concentrations were quantified using GC-MS (Shimadzu-QP2010 SE, Japan) as described previously [15]. SCFA analysis was performed using a MEGA-WAX MS column with helium as the carrier gas at a flow rate of 3 mL/min. Sample preparation, chromatographic conditions, and compound identification followed established protocols [15].

Ammonia nitrogen (NH_3_–N) concentration was determined spectrophotometrically using the phenol–hypochlorite method described earlier [16]. Absorbance was measured at 630 nm using a Shimadzu UV-1800 portable spectrophotometer.

After *in vitro* fermentation, samples were separated using Whatman™ 41 filter papers (Thermo Fisher Scientific, USA, Cat. No. 1441-125). Solid residues were incubated with pepsin-HCl (2 g L^-^¹ pepsin and 17.8 mL·L^-^¹ HCl) at 39°C for 72 h. Residues were filtered and oven-dried at 105°C for 24 h to determine digestibility [17].

### Partitioning factor and microbial protein estimation

PF was calculated as the ratio of dry matter degraded to total gas production and used as an indicator of microbial biomass synthesis efficiency [18]. Microbial protein synthesis was estimated based on OMD using the following equation described previously [19]:

Microbial protein (g/kg organic matter digestibility) = OMD (g) × 19.3 × 6.25

### Gas production kinetics

Total gas production kinetics were evaluated using Ørskov’s exponential model described previously [20, 21]:

p = a + b (1 − e^-ct^),

where p represents cumulative gas production, a is gas from the soluble fraction, b is gas from the insoluble fraction, c is the gas production rate constant, and t is incubation time.

### Statistical analysis

Data were analyzed using analysis of variance (ANOVA) in IBM SPSS version 22.0. The experimental design was a completely randomized design comprising 14 macroalgae treatments with five replicates per treatment. The statistical model applied was:

Yij = μ + τi + εij

Where:

Yij= observation from treatment i, replicate j

μ = overall mean

𝜏i = treatment effect

𝜀ij = random error

Differences among treatment means were evaluated using Duncan’s post-hoc test. Statistical analysis followed established procedures described previously [22].

## RESULTS AND DISCUSSION

### *In vitro* rumen fermentation profiles of macroalgae

Total gas production differed significantly among the macroalgal species tested. *B. forbesii* generated substantially higher total gas than *T. ornata* (p < 0.05; Table 3) and exhibited the highest overall gas production (p < 0.01; Table 3). Most other species, including *Sargassum* sp., *P. palmata*, and *G. gigas*, produced intermediate gas volumes, with no significant differences among them (p > 0.05; Table 3).

**Table 3:** *In vitro* total gas production in the rumen and enteric methane emission of several macroalgae species.

Macroalgae species	Total gas production (mL)	Estimated methane production (mM)	Estimated methane production (mM/g incubated DM)	Estimated methane production/total gas production (mM/mL)
Brown seaweeds				
*Padina* sp.	17.30 ± 6.92^ab^	23.87 ± 6.22^cd^	47.72 ± 12.44^ef^	1.81 ± 1.36^ab^
*Sargassum* sp.	30.83 ± 9.19^abc^	1.87 ± 0.08^a^	3.75 ± 0.17^a^	0.06 ± 0.03^a^
*Turbinaria ornata*	10.94 ± 7.75^a^	11.88 ± 7.94^abc^	23.76 ± 15.88^abc^	1.58 ± 1.30^bc^
Green seaweeds				
*Boergesenia forbesii*	54.75 ± 4.84^c^	15.32 ± 10.94^abc^	30.63 ± 21.87^bcdef^	0.50 ± 0.62^ab^
*Caulerpa racemosa*	19.74 ± 9.53^ab^	6.66 ± 3.40^ab^	13.31 ± 6.79^ab^	0.59 ± 0.72^bc^
*Ulva lactuca*	31.91 ± 15.90^abc^	22.63 ± 14.40^cd^	45.25 ± 28.79^def^	1.43 ± 1.85^abc^
Red seaweeds				
*Eucheuma cottonii*	19.36 ± 9.90^ab^	19.11 ± 7.76^bcd^	38.20 ± 15.52^cde^	1.93 ± 2.57^abc^
*Eucheuma spinosum*	18.74 ± 13.60^abc^	14.03 ± 7.87^abc^	28.05 ± 15.73^abcde^	0.68 ± 0.39^abc^
*Gelidium* sp.	18.50 ± 8.74^ab^	11.11 ± 2.74^abc^	22.22 ± 5.48^abcd^	0.71 ± 0.32^ab^
*Gracilaria coronopifolia*	23.89 ± 13.96^abc^	11.31 ± 8.29^abc^	22.62 ± 16.58^abcde^	0.92 ± 0.62^c^
*Gracilaria gigas*	35.16 ± 16.91^bc^	20.82 ± 8.70^bcd^	41.63 ± 17.40^cdef^	0.96 ± 1.02^abc^
*Gracilaria verrucosa*	29.23 ± 12.54^abc^	21.51 ± 8.06^bcd^	43.01 ± 16.12^cdef^	0.92 ± 0.62^abc^
*Halymenia durvillei*	14.07 ± 6.40^ab^	31.80 ± 7.29^d^	63.58 ± 14.58^f^	3.11 ± 2.54^ab^
*Palmaria palmata*	21.89 ± 9.84^ab^	13.46 ± 3.47^abc^	26.91 ± 6.93^abcde^	0.75 ± 0.40^ab^
p-value	0.01	0.01	0.01	0.05

Means with different superscripts within a column are significantly different (p < 0.01 and p < 0.05). Values are presented as mean ± standard deviation. DM = dry matter.

Means with different superscripts within a column are significantly different (p < 0.01 and p < 0.05). Values are presented as mean ± standard deviation. DM = dry matter.

This pattern indicates that *B. forbesii* contains highly fermentable organic constituents, enabling extensive degradation by rumen microorganisms. In contrast, *T. ornata* showed consistently low gas output, reflecting limited microbial degradation, most likely due to higher fiber or mineral content that restricts microbial access.

*B. forbesii* and *G. gigas* demonstrated comparatively high gas production, further supporting their enhanced fermentability. Species such as *E. spinosum* and *H. durvillei* provided highly digestible biomass, making them particularly suitable as ruminant feed ingredients. Their substantially higher digestibility distinguishes them from other species. Conversely, *T. ornata*, *Gelidium* sp., and *G. coronopifolia* exhibited markedly lower digestibility, likely attributable to elevated fiber or mineral levels that inhibit microbial breakdown.

The efficiency of converting digested dry matter into microbial biomass (rather than gas) was broadly similar across species. This consistency suggests that the primary differences lie in how each macroalga partitions metabolized nutrients, some favoring microbial growth, others directing more toward gas production.

Among all species evaluated, *Sargassum* sp. recorded the lowest estimated methane concentration and methane output per unit of incubated dry matter, underscoring its strong potential as a methane-mitigation feed resource. By contrast, *H. durvillei* produced the highest methane yield per unit of gas, indicating a less environmentally favorable fermentation profile.

### Overall implications for species selection

These findings emphasize the critical importance of species-specific selection when incorporating macroalgae into ruminant diets. Highly fermentable and digestible species (e.g., *B. forbesii*, *E. spinosum*, and *H. durvillei*) offer excellent potential for improving nutrient availability and fermentation efficiency. Less fermentable species (e.g., *T. ornata*, *Gelidium* sp.) may be better suited for targeted applications, such as partial diet inclusion where methane mitigation or other bioactive effects are prioritized over maximum digestibility.

Careful selection based on these fermentation characteristics allows optimization of macroalgae as sustainable feed components, balancing nutrient supply, microbial efficiency, and environmental benefits (particularly methane reduction) in ruminant production systems.

### Methane production and mitigation potential

Methane production (expressed as millimolar concentration, mM) varied substantially among the macroalgal species (p < 0.01; Table 3). *H. durvillei* produced the highest methane concentration, significantly exceeding that of *Sargassum* sp. and *C. racemosa* (p < 0.01). In contrast, *Sargassum* sp. exhibited the lowest methane production per unit of incubated dry matter (DM) and the lowest overall methane output compared to the other species (p < 0.01; Table 3). This resulted in marked reductions in enteric methane emissions, approximately 71.86% relative to certain species and up to 94.11% when compared directly with *H. durvillei*.

The methane-to-gas production ratio was also strongly affected (p < 0.01). Overall, the highly significant differences (p < 0.01) demonstrated that macroalgae species differ markedly in their fermentation characteristics and methane emission potential.

*Sargassum* sp. displayed a distinctive fermentation profile characterized by substantial methane reduction despite moderate to high total gas production (Table 3). Among all species evaluated, it recorded the lowest methane concentration, methane yield per gram of dry matter, and methane output per unit of gas produced.

*C. racemosa* and *U. lactuca* exhibited lower methane-to-gas ratios, indicating a more favorable profile for methane suppression. By comparison, *H. durvillei* showed a higher methane content in its gas production. An ideal feed additive or compound for suppressing methanogenesis in ruminants would increase propionate production (a glucogenic volatile fatty acid) while simultaneously reducing CH_4_ output.

Among the species tested, *Sargassum* sp. and *C. racemosa* emerged as the most promising candidates for methane mitigation. They combine moderate fermentation performance with substantially reduced methane emissions, making them particularly valuable for developing sustainable ruminant feeding strategies aimed at lowering enteric methane production.

These results highlight the potential of selected macroalgae to serve as targeted functional feed additives, offering a practical approach to enteric methane reduction without severely compromising overall fermentation or nutrient supply in ruminant diets.

### Methane inhibition mechanisms and species-specific effects

High methane inhibition by *Sargassum* sp. was manifested by a reduction in volatile fatty acid (VFA) content, resulting in a measurably higher rumen pH. This effect is most likely attributable to bioactive secondary metabolites in *Sargassum* sp., including polyphenols and halogenated compounds, which inhibit methanogenic archaea. Comparable effects have been reported in other CH_4_ inhibition studies [23] and in *in vivo* trials where sheep were supplemented with *A. taxiformis* [24].

In contrast, *H. durvillei* and *Padina* sp. produced elevated methane levels. This characteristic may restrict their direct use in enteric methane reduction strategies unless combined with established inhibitory agents.

These species-specific differences highlight the importance of targeted assessments when selecting macroalgae for sustainable ruminant feed formulations and methane mitigation programs.

### Variation in estimated methane reduction

**Figure 2:**
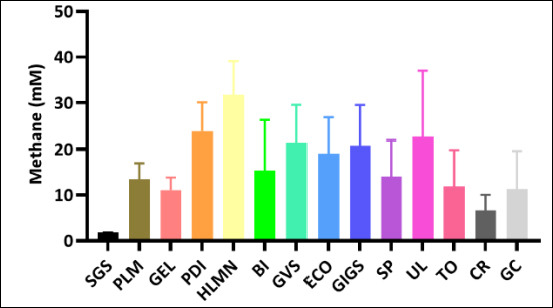
Reduction of estimated methane production (mM) across macroalgae species. Reduction of estimated methane production between macroalgae (mM). SGS = *Sargassum* sp., PLM = *Palmaria palmata*, GEL = *Gelidium* sp., PDI = *Padina* sp., HLMN = *Halymenia durvillei*, BI= *Boergesenia forbesii*, GVS = *Gracilaria verrucosa*, ECO = *Eucheuma cottonii*, GIGS = *Gracilaria gigas*, SP = *Eucheuma spinosum*, UL = *Ulva lactuca*, TO = *Turbinaria ornata*, CR = *Caulerpa racemosa*, GC = *Gracilaria coronopifolia*.

Estimated methane production decreased to varying degrees across the macroalgal species evaluated. Distinct differences were observed among treatments, revealing substantial variation in methane suppression potential. Several species achieved significantly lower approximated methane production than others, while some showed only modest reductions. Overall, the extent of methane mitigation was highly species-dependent (Figure 2)

### Rumen pH and fermentation stability

*In vitro* rumen pH varied only marginally among treatments but remained within the normal physiological range (p < 0.05; Table 4). All values stayed within the optimal range of 6.5–7.2. *Sargassum* sp., *Gelidium* sp., and *P. palmata* exhibited comparatively higher pH levels, which are favorable for fiber-degrading bacteria. Conversely, *C. racemosa* showed the lowest pH values, though still acceptable.

**Table 4:** *In vitro* rumen pH, ammonia, and partitioning factor of several macroalgae species.

Macroalgae species	pH	Ammonia (N-NH_3_) (mg/dL)	Partitioning Factor (mg DMD/mL gas)	Microbial protein (g/kg OMD)
Brown seaweeds				
*Padina* sp.	6.98 ± 0.05^abc^	55.63 ± 29.24	17.30 ± 6.92	48.22 ± 35.27^abcd^
*Sargassum* sp.	7.04 ± 0.11^c^	56.23 ± 15.67	30.83 ± 9.19	16.19 ± 3.82^a^
*Turbinaria ornata*	6.97 ± 0.07^abc^	56.27 ± 16.56	10.94 ± 7.75	14.30 ± 7.83^a^
Green seaweeds				
*Boergesenia forbesii*	6.92 ± 0.10^abc^	63.59 ± 23.76	54.75 ± 4.84	73.89 ± 41.35^bcde^
*Caulerpa racemosa*	6.83 ± 0.07^a^	48.45 ± 18.26	19.74 ± 9.53	27.94 ± 8.80^ab^
*Ulva lactuca*	6.87 ± 0.06^ab^	58.34 ± 19.36	31.91 ± 15.90	87.70 ± 10.31^de^
Red seaweeds				
*Eucheuma cottonii*	6.95 ± 0.08^abc^	59.84 ± 20.05	19.36 ± 9.86	62.63 ± 12.82^bcde^
*Eucheuma spinosum*	6.95 ± 0.03^abc^	41.16 ± 24.44	18.75 ± 13.59	111.75 ± 30.93^e^
*Gelidium* sp.	7.01 ± 0.06^bc^	56.45 ± 19.13	18.50 ± 8.74	15.90 ± 5.31^a^
*Gracilaria coronopifolia*	6.95 ± 0.06^abc^	49.92 ± 12.76	23.89 ± 13.96	11.65 ± 2.99^ab^
*Gracilaria gigas*	6.96 ± 0.07^abc^	55.20 ± 32.32	35.16 ± 16.91	62.81 ± 7.71^bcde^
*Gracilaria verrucosa*	7.01 ± 0.11^bc^	55.99 ± 24.20	29.23 ± 12.54	60.87 ± 13.05^bcde^
*Halymenia durvillei*	6.94 ± 0.15^abc^	75.84 ± 31.21	14.07 ± 6.40	76.96 ± 20.53^cde^
*Palmaria palmata*	7.01 ± 0.11^bc^	54.52 ± 9.02	21.89 ± 9.84	44.41 ± 8.53^abc^
p-value	<0.05	0.068	0.171	0.01

Means with different superscripts within a column are significantly different (p < 0.01 and p < 0.05). Values are presented as mean ± standard deviation. DMD = dry matter digestibility, OMD = organic matter digestibility.

Certain macroalgae may exert mild buffering or acidifying effects on the rumen environment. However, rumen fermentation parameters remained near neutrality across all treatments (Table 4), indicating that macroalgae inclusion did not cause harmful ruminal conditions.

### Ammonia concentrations and microbial protein synthesis

Ammonia (NH_3_) concentrations showed no significant differences among macroalgal treatments (p > 0.05; Table 4). Nevertheless, *H. durvillei* recorded the highest ammonia levels, suggesting rapid and extensive protein degradation. *E. spinosum* exhibited the lowest ammonia concentrations, implying more efficient nitrogen incorporation and utilization by rumen microorganisms.

The elevated ammonia in *H. durvillei* may reflect either reduced microbial nitrogen assimilation or accelerated protein breakdown rates. Species differences in protein degradability, likely influenced by protein content and secondary metabolites, appear to drive these patterns, as reflected in the ammonia levels produced during incubation.

Ammonia concentrations across all species were sufficient to support microbial growth, despite some numerical variation. Microbial protein synthesis differed significantly among treatments, with higher production observed in *U. lactuca*, *E. spinosum*, and *H. durvillei*. These results demonstrate that macroalgae vary in their capacity to synchronize energy and nitrogen release, which directly influences microbial efficiency and fermentation outcomes.

### Partitioning factor and microbial efficiency

The partitioning factor (PF; Table 4; p > 0.05) showed wide variation among the macroalgal substrates. PF is calculated as the ratio of truly degraded organic matter (mg) to gas produced (mL). Higher PF values indicate greater fermentation efficiency, with a larger proportion of metabolized dry matter retained as microbial biomass rather than lost as gas.

Microbial protein production differed significantly among treatments (p < 0.05). Certain macroalgae strongly supported high microbial protein synthesis; for example, one sample yielded 111.75 g of microbial protein per kg of organic matter degraded (OMD), demonstrating excellent microbial efficiency.

*B. forbesii* exhibited the highest PF, reflecting superior conversion of degraded substrate into microbial biomass relative to gas production. In contrast, *T. ornata* and *H. durvillei* had the lowest PF values, indicating less efficient incorporation of degraded material into microbial mass. Microbial protein synthesis reached its maximum in *E. spinosum* and *U. lactuca*, whereas *Gelidium* sp. and *Sargassum* sp. produced the lowest yields, possibly due to inhibitory secondary compounds or limited nutrient availability.

### Ammonia dynamics and protein fractions

Decreases in ammonia concentration generally reflect increased incorporation of ammonia-nitrogen (NH_3_-N) into microbial protein by rumen microorganisms. This process can be constrained by reduced proteolysis or insufficient fermentable carbohydrate availability [25].

Different macroalgae species contain varying proportions of rumen-degradable protein (RDP; protein broken down in the rumen to supply ammonia and peptides for microbial growth) and rumen-undegradable protein (RUP; bypass protein that escapes rumen fermentation and is digested in the small intestine for direct benefit to the host animal) [26, 27]. Further detailed characterization of RDP and RUP fractions in macroalgae is warranted. Optimizing RUP content could significantly improve feed efficiency, body weight gain, and milk production in ruminants. Secondary metabolites such as tannins, saponins, and flavonoids are known to influence the RDP/RUP balance by binding proteins or modulating microbial activity.

High NH_3_-N concentrations typically indicate poor rumen ammonia absorption, excessive deamination of amino acids, or slow microbial uptake and metabolism of ammonia [28]. In this study, ammonia levels remained adequate to sustain microbial growth across all species, despite some numerical variation.

### Implications for ruminant nutrition and feed formulation

PF and microbial protein synthesis are critical indicators of how effectively rumen microorganisms utilize feed substrates during fermentation. Higher PF values signify better nutrient partitioning toward microbial biomass production, thereby enhancing the supply of microbial protein to the host ruminant and improving overall nitrogen efficiency.

The results demonstrate that macroalgae differ markedly in their capacity to provide fermentable energy and available nitrogen to support rumen microbial growth. Some species promote high microbial protein synthesis and efficient substrate utilization, while others are less effective. Selective inclusion of macroalgae with favorable PF and high microbial protein yields can therefore enhance rumen microbial efficiency, increase protein delivery to the animal, and contribute to more sustainable and productive ruminant feeding systems. It is imperative to exercise caution when selecting species in order to optimize these advantages.

These findings reinforce the value of macroalgae as a diverse resource in ruminant nutrition, where strategic use of high-PF, protein-efficient species can optimize microbial protein supply and nitrogen utilization while minimizing environmental impacts.

### VFA production and fermentation pathways

Decreased VFA production in *Sargassum* sp. suggests that fermentation was selectively suppressed rather than simply inefficient. Brown macroalgae, including *Sargassum* sp., contain bioactive secondary metabolites such as phlorotannins and other phenolic compounds. These compounds can selectively inhibit specific rumen microbial populations, particularly methanogenic archaea and protozoa. This targeted inhibition likely reduced hydrogen availability for methanogenesis while simultaneously limiting overall VFA accumulation.

*Sargassum* sp. exhibited a high partitioning factor (PF; Table 4), indicating efficient conversion of degraded substrate into microbial biomass relative to gas production. However, its microbial protein yield was among the lowest, suggesting that microbial growth was limited in scale but not necessarily impaired in substrate utilization efficiency. Thus, methane mitigation by *Sargassum* sp. appears to result from microbial community modulation rather than reduced digestibility. Fermentation pathways were diverted away from methanogenesis, even though this led to lower overall VFA production, consistent with partial inhibition of certain rumen microbial activities.

In contrast, *H. durvillei* displayed vigorous fermentation, as evidenced by the highest total VFA concentration among all species (127.51 mM; Table 5), together with elevated acetate and propionate production. *H. durvillei* also recorded the highest ammonia concentration (75.84 mg/dL; Table 4), reflecting substantial protein degradation and robust microbial activity. These parameters collectively confirm that *H. durvillei* provided abundant fermentable substrates and nitrogen to support rumen microorganisms.

**Table 5:** *In vitro* rumen total VFA, short-chain fatty acid, and acetate-to-propionate (A/P) ratio of several macroalgae species.

Macroalgae species	Total VFA (mM)	Acetate (mM)	Propionate (mM)	Butyrate (mM)	Valerate (mM)	iso-Butyrate (mM)	iso-Valerate (mM)	A/P
Brown seaweeds								
*Padina* sp.	99.15 ± 23.49^ab^	55.16 ± 14.28^ab^	28.49 ± 6.36^abc^	7.09 ± 1.34^ab^	0.95 ± 0.18^b^	2.81 ± 0.62^abc^	4.66 ± 0.96^ab^	1.92 ± 0.12^cd^
*Sargassum* sp.	45.08 ± 24.22^a^	23.98 ± 12.51^a^	13.03 ± 7.33^ab^	3.40 ± 2.01^ab^	0.41 ± 0.31^ab^	1.67 ± 0.79^abc^	2.59 ± 1.32^ab^	1.87 ± 0.08^bcd^
*Turbinaria ornata*	80.31 ± 73.24^ab^	46.56 ± 43.57^ab^	22.21 ± 20.05^abc^	5.28 ± 5.05^ab^	1.05 ± 0.70^ab^	2.19 ± 1.44^abc^	3.44 ± 2.53^ab^	2.02 ± 0.16^cd^
Green seaweeds								
*Boergesenia forbesii*	95.91 ± 66.76^ab^	43.66 ± 30.86^ab^	38.01 ± 26.36^c^	6.39 ± 4.57^ab^	1.36 ± 0.19^b^	2.68 ± 1.45^abc^	4.36 ± 2.80^ab^	1.12 ± 0.10^a^
*Caulerpa racemosa*	30.31 ± 17.09^a^	15.84 ± 7.77^a^	9.24 ± 5.24^a^	2.17 ± 1.72^a^	0.27 ± 0.10^a^	1.15 ± 0.94^a^	1.75 ± 1.46^a^	1.83 ± 0.29^bcd^
*Ulva lactuca*	86.53 ± 59.94^ab^	54.04 ± 34.11^ab^	29.28 ± 16.01^abc^	6.23 ± 3.08^ab^	0.90 ± 0.42^b^	2.74 ± 1.20^abc^	4.08 ± 1.88^ab^	1.81 ± 0.19^bcd^
Red seaweeds								
*Eucheuma cottonii*	86.27 ± 31.07^ab^	45.24 ± 18.36^ab^	26.11 ± 9.13^abc^	6.35 ± 1.80^ab^	0.88 ± 0.35^ab^	2.85 ± 0.55^bc^	4.84 ± 1.12^b^	1.70 ± 0.14^bc^
*Eucheuma spinosum*	61.77 ± 33.30^ab^	32.87 ± 18.20^ab^	18.20 ± 9.74^abc^	4.49 ± 2.66^ab^	0.66 ± 0.36^ab^	2.24 ± 0.86^abc^	3.44 ± 1.74^ab^	1.81 ± 0.17^bcd^
*Gelidium* sp.	47.95 ± 11.23^a^	25.53 ± 6.33^a^	13.51 ± 3.42^ab^	3.67 ± 0.83^ab^	0.50 ± 0.09^ab^	1.73 ± 0.31^abc^	3.01 ± 0.45^ab^	1.89 ± 0.07^cd^
*Gracilaria coronopifolia*	51.87 ± 28.17^ab^	27.66 ± 17.24^a^	16.35 ± 7.69^abc^	3.45 ± 2.32^ab^	0.65 ± 0.49^ab^	1.56 ± 0.62^ab^	2.21 ± 1.30^ab^	1.72 ± 0.58^bcd^
*Gracilaria gigas*	97.85 ± 35.65^ab^	50.44 ± 20.63^ab^	31.66 ± 10.60^abc^	7.45 ± 2.26^b^	0.96 ± 0.38^b^	2.73 ± 0.72^abc^	4.62 ± 1.33^ab^	1.56 ± 0.14^b^
*Gracilaria verrucosa*	95.60 ± 34.62^ab^	50.87 ± 18.63^ab^	29.46 ± 10.45^abc^	7.15 ± 3.04^ab^	0.84 ± 0.45^ab^	2.75 ± 0.82^abc^	4.52 ± 1.52^ab^	1.72 ± 0.13^bcd^
*Halymenia durvillei*	127.51 ± 24.25^b^	73.85 ± 16.63^b^	36.13 ± 6.43^bc^	8.11 ± 1.24^b^	1.02 ± 0.21^b^	3.30 ± 0.32^c^	5.09 ± 0.57^b^	2.03 ± 0.16^d^
*Palmaria palmata*	61.99 ± 15.02^ab^	31.65 ± 8.30^a^	18.39 ± 4.46^abc^	4.78 ± 1.08^ab^	0.71 ± 0.25^ab^	2.45 ± 0.35^abc^	4.02 ± 0.85^ab^	1.71 ± 0.10^bcd^
p-value	0.01	0.012	0.012	0.014	0.057	0.012	0.015	0.012

Means with different superscripts within a column are significantly different (*p* < 0.01 and p < 0.05). Values are presented as mean ± standard deviation. VFA = volatile fatty acid; A/P = acetate-to-propionate ratio.

### Species comparisons in VFA profiles

*C. racemosa* and *Sargassum* sp. differed significantly from *H. durvillei*, which produced the highest total VFA levels (p < 0.01; Table 5). Total VFA and propionate concentrations were higher in *H. durvillei* and *B. forbesii* than in the other species (p < 0.01; Table 5). *H. durvillei* generated the highest acetate and butyrate levels, significantly exceeding those of the low-yielding *C. racemosa* (p = 0.012). *B. forbesii* and *H. durvillei* were also among the highest in propionate production, with significant differences from *C. racemosa* (p < 0.01; Table 5).

*H. durvillei* showed the highest isobutyrate and isovalerate concentrations, which were significantly greater than those in *C. racemosa* (0.27 ± 0.10 mM and 1.75 ± 1.46 mM, respectively; p < 0.01; Table 5). No significant difference was observed in valerate production (p = 0.057), suggesting that fermentation pathways leading to this acid were relatively consistent across species.

These results highlight distinct fermentation profiles among the macroalgae, with *Sargassum* sp. favoring methane suppression through selective microbial inhibition and *H. durvillei* promoting high VFA and ammonia output indicative of intense, non-selective fermentation. The contrasting patterns underscore the potential of *Sargassum* sp. as a methane-mitigating additive while illustrating that high-fermentability species like *H. durvillei* may require careful inclusion to avoid excessive methane emissions.

### VFA profiles and implications for methane mitigation

Methane mitigation in *Sargassum* sp. coincided with low overall VFA production (45.08 mM; Table 5) and comparatively low concentrations of acetate, propionate, and butyrate. These observations suggest that fermentative activity was somewhat restricted, despite the species having a relatively high non-fiber extract content (57.94%) and moderate crude protein levels (Table 2).

The A/P ratio varied significantly among species (p = 0.012). *B. forbesii* exhibited the lowest A/P ratio (1.12 ± 0.10), which was substantially lower than that of *H. durvillei* (2.03 ± 0.16) and *Padina* sp. (1.92 ± 0.12). Statistically significant differences were observed among macroalgal species for total VFA, acetate, propionate, butyrate, isobutyrate, isovalerate, and A/P ratio, while valerate showed no significant difference (p = 0.057).

Total VFA concentration was highest in *H. durvillei*, followed by *Padina* sp. and *G. gigas*, indicating greater fermentative activity in these species. In contrast, *C. racemosa* and *Sargassum* sp. produced the lowest total VFA levels, reflecting reduced fermentability. *B. forbesii* and *H. durvillei* achieved the highest propionate concentrations, a favorable profile for methane mitigation, as propionate serves as an alternative hydrogen sink that competes with methanogenesis. *C. racemosa* consistently showed the lowest levels of major VFAs.

These findings align with previous research on brown macroalgae. For example, three brown species (*U. pinnatifida* [UPIN], *S. fusiforme* [SFUS], and *S. fulvellum* [SFUL]) increased rumen VFA production when included at 1% DM [29]. Phlorotannins in brown macroalgae have been shown to occasionally exert antimicrobial and antimethanogenic effects in ruminants. In that study, CH_4_ production was reduced at 1% DM inclusion in SFUS, but not in UPIN or SFUL. Additionally, after 48 h of incubation, CH_4_ production was significantly reduced when 0.25 mg/mL timothy hay was added to macroalgal extracts [29].

### NSP and hydrogen sink manipulation

Characterizing the fermentability of individual non-starch polysaccharide components is essential when incorporating macroalgae into ruminant diets. Intact macroalgae rich in non-starch polysaccharides may function as wholesome, fiber-based feed ingredients [30].

Rumen methane production arises from syntrophic interactions between H_2_-producing microbes and H_2_-oxidizing methanogens [31]. Manipulating the hydrogen sink is a key strategy for reducing CH_4_ emissions. Becker *et al*. [32] proposed that flavan-3-ol compounds, such as (+)-catechin, could serve as alternative H_2_ sinks for methane precursors without compromising VFA production. However, the present results appear somewhat conflicting: while *Sargassum* sp. showed methane suppression with low VFA output, certain brown macroalgae (e.g., SFUS and SFUL in prior work) exhibited higher VFA production alongside methane reduction, suggesting species-specific mechanisms that warrant further investigation.

Overall, these VFA profiles reinforce the potential of selected macroalgae, particularly those with favorable propionate promotion or selective inhibition (e.g., *Sargassum* sp. and *B. forbesii*), for methane mitigation, while highlighting the need to balance fermentability, nutrient supply, and environmental benefits in ruminant feed design.

### Correlations between fermentation end-products and methane production

**Figure 3:**
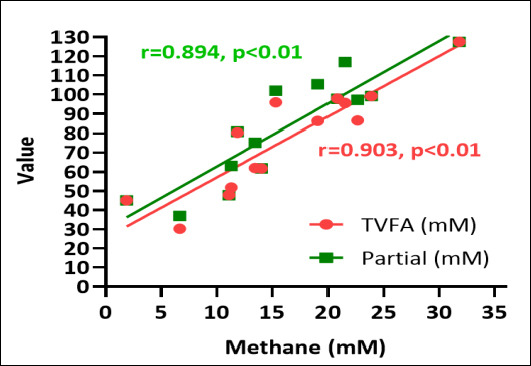
Correlation between estimated methane production (mM), total volatile fatty acid (TVFA), and individual short-chain fatty acid components (mM).

Figure 3 illustrates the correlations between estimated methane production, TVFA, and individual SCFA components. Estimated methane production showed clear associations with both TVFA and specific SCFA fractions. Negative correlations were observed between methane production and propionate concentration, whereas positive correlations existed between methane production and acetate concentration. These relationships confirm consistent links between the distribution of fermentation end-products and methano-genesis: acetate-dominant pathways promote higher methane output, while propionate-promoting pathways divert hydrogen away from methanogens, reducing CH_4_ formation.

Variations in A/P ratios across species reflect changes in hydrogen utilization directly linked to methane generation. Lower A/P ratios, as observed in *B. forbesii* and *C. racemosa*, are typically associated with decreased methane production, supporting the methane mitigation potential of certain green macroalgae. A shift toward higher relative propionate favors a more glucogenic fermentation profile, improving energy efficiency for the host ruminant and lowering enteric methane emissions. Dietary macroalgae supplementation has previously been shown to numerically decrease acetate (−1.9%) and increase propionate (+4.7%), contributing to a reduced A/P ratio [33].

### Species-specific VFA profiles and fermentation patterns

*H. durvillei* exhibited superior fermentative output across most VFA parameters, producing the highest total VFA, acetate, propionate, butyrate, isobutyrate, and isovalerate concentrations. In contrast, *C. racemosa* consistently showed the lowest values for these parameters. These marked differences highlight how macroalgal species profoundly influence rumen fermentation responses.

Elevated TVFA concentrations in *H. durvillei*, *Padina* sp., and certain *Gracilaria* species indicate greater overall fermentative activity, whereas *Sargassum* sp. and *C. racemosa* displayed the lowest TVFA levels, reflecting limited fermentability. *B. forbesii* and *H. durvillei* achieved the highest propionate concentrations, a beneficial profile for methane mitigation due to propionate’s role as a competing hydrogen sink.

**Figure 4:**
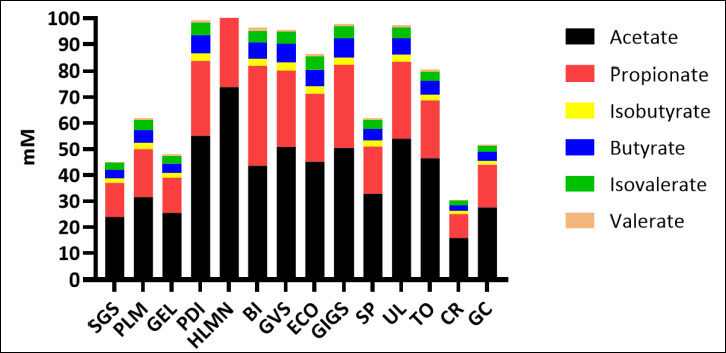
Comparative profile of partial volatile fatty acids (short-chain fatty acids) (mM) across macroalgae species. SGS = Sargassum sp., PLM = *Palmaria palmata*, GEL = *Gelidium* sp., PDI = Padina sp., HLMN = *Halymenia durvillei*, BI= *Boergesenia forbesii*, GVS = *Gracilaria verrucosa*, ECO = *Eucheuma cottonii*, GIGS = *Gracilaria gigas*, SP = *Eucheuma spinosum*, UL = *Ulva lactuca*, TO = *Turbinaria ornata*, CR = *Caulerpa racemosa*, GC = *Gracilaria coronopifolia*.

**Figure 5:**
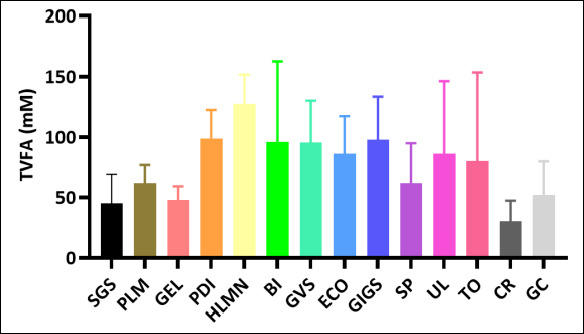
Comparison of total volatile fatty acid (TVFA) profile (mM) across macroalgae species. SGS = *Sargassum* sp., PLM = *Palmaria palmata*, GEL = *Gelidium* sp., PDI = Padina sp., HLMN = *Halymenia durvillei*, BI= *Boergesenia forbesii*, GVS = G*racilaria verrucosa*, ECO = *Eucheuma cottonii*, GIGS = *Gracilaria gigas*, SP = *Eucheuma spinosum*, UL = *Ulva lactuca*, TO = *Turbinaria ornata*, CR = *Caulerpa racemosa*, GC = *Gracilaria coronopifolia*.

The relative proportions of individual SCFAs (acetate, propionate, butyrate) varied considerably among species (Figure 4), with some macroalgae favoring acetate- or butyrate-dominant patterns and others promoting elevated propionate. TVFA production showed substantial variability across treatments (Figure 5), confirming species-dependent differences in fermentation intensity.

### Mechanistic insights and implications

Species with acetate-dominant fermentation patterns generally produced more methane, reinforcing the strong stoichiometric relationship between VFA profiles and methanogenesis. Differences in branched-chain VFA and ammonia concentrations further indicate variation in protein degradation rates and microbial growth efficiency, consistent with the observed disparities in microbial protein synthesis.

Differences in ruminal degradability among macroalgae are likely attributable to variations in cell wall polysaccharides [34]. Previous studies [4, 34] have reported CH_4_ reductions without significant losses in digestibility [4], aligning with the 24 h *in vitro* VFA yields observed here. Elevated butyrate levels in some treatments may be linked to increased abundance of certain poorly characterized *Lachnospiraceae* genera (e.g., *Lachnospiraceae* AC2004, FCS020, NK4A136) that co-occur with potent methane inhibitors such as *A. taxiformis*. These bacteria may help sustain fiber degradation even when cellulolytic populations are reduced [35].

Overall, these VFA profiles, correlations, and species-specific patterns emphasize that macroalgae exert distinct effects on rumen fermentation pathways. Targeted selection of species that promote propionate production (e.g., *B. forbesii*, *H. durvillei*) or selectively inhibit methanogens (e.g., *Sargassum* sp.) offers strong potential for enteric methane mitigation while preserving nutrient utilization in sustainable ruminant diets.

### Digestibility and nutrient availability across macroalgal species

Reduced propionate production may be attributed to the inhibition of many key succinate-utilizing bacteria following *A. taxiformis* supplementation. Notably, *A. taxiformis* supplementation expanded the populations of genera containing putative succinate producers, i.e., *Succinivibrio* (stable phase) and *Ruminobacter* (intermediate and stable phases). Ruminal succinate may play roles beyond propionate production in *A. taxiformis* supplementation [35]. Among the macroalgae evaluated, *H. durvillei* and *E. spinosum* demonstrated the highest IVOMD (p < 0.01; Table 6). IVDMD of *E. spinosum* and *H. durvillei* was significantly higher than that of *T. ornata* and *Gelidium* sp. (p < 0.01; Table 6). Mid-range digestibilities were recorded in *B. forbesii* and *G. gigas*, confirming substantial interspecies variation. *E. spinosum* and *H. durvillei* exhibited the highest IVOMD values, surpassing those of *T. ornata* and *G. coronopifolia* (p < 0.01; Table 6).

**Table 6:** *In vitro* rumen dry matter and organic matter digestibility of several macroalgae species.

Macroalgae species	IVDMD (%)	IVOMD (%)
Brown seaweeds		
*Padina* sp.	31.60 ± 12.49^ab^	39.98 ± 29.24^abcd^
*Sargassum* sp.	26.98 ± 2.31^a^	13.42 ± 3.17^ab^
*Turbinaria ornata*	24.69 ± 6.08^a^	11.85 ± 6.49^a^
Green seaweeds		
*Boergesenia forbesii*	52.00 ± 4.57^cd^	46.03 ± 4.57^cde^
*Caulerpa racemosa*	47.07 ± 4.11^abc^	23.17 ± 7.30^abc^
*Ulva lactuca*	77.93 ± 7.94^de^	72.70 ± 8.55^cd^
Red seaweeds		
*Eucheuma cottonii*	60.21 ± 9.70^cd^	51.92 ± 10.63^cd^
*Eucheuma spinosum*	79.85 ± 6.40^e^	74.60 ± 9.63^e^
*Gelidium* sp.	21.78 ± 5.15^a^	13.19 ± 4.40^ab^
*Gracilaria coronopifolia*	20.85 ± 1.31^ab^	9.66 ± 2.48^abc^
*Gracilaria gigas*	53.51 ± 5.30^bc^	52.07 ± 6.39^cd^
*Gracilaria verrucosa* Sidoarjo	52.90 ± 10.55^bc^	50.46 ± 10.82^bcd^
*Halymenia durvillei*	81.72 ± 11.67^de^	69.53 ± 19.54^de^
*Palmaria palmata*	44.19 ± 6.64^abc^	36.82 ± 7.07^abcd^
p-value	0.011	0.01

Means with different superscripts within a column are significantly different (p < 0.01 and p < 0.05). Values are presented as mean ± standard deviation. IVDMD = *In vitro* dry matter digestibility; IVOMD = *In vitro* organic matter digestibility.

The highest IVDMD and IVOMD were observed in *H. durvillei*, *E. spinosum*, and *U. lactuca*, indicating enhanced nutrient accessibility for rumen microorganisms. Conversely, *Gelidium* sp. and *G. coronopifolia* showed the lowest digestibility values, likely due to high fiber content or resistant polysaccharides that limit microbial degradation. *P. palmata*, *Eucheuma cottonii*, and *G. gigas* displayed moderately high digestibility. The consistent trends in IVDMD and IVOMD highlight distinct fiber and polysaccharide structures among macroalgae species, which directly affect ruminal microbial degradation.

Red and green macroalgae, including *E. spinosum*, *H. durvillei*, and *U. lactuca*, demonstrated favorable ruminal degradability and energy accessibility. In contrast, lower digestibility in *Gelidium* sp. and certain *Gracilaria* species may be linked to elevated fiber or complex polysaccharides resistant to breakdown. These findings indicate that high digestibility, methane reduction, and fermentation efficiency do not always align and must be evaluated together (Table 6).

In other studies, *P. palmata* yielded the highest values (4.34 mmol VFA and 833 g/kg in situ DM degradability). Nutrient digestibility in various gastrointestinal fractions was adversely affected by A. nodosum inclusion [36]. Alaria fractions may reduce OMD, paralleling the inhibitory effects of *A. taxiformis* on CH_4_ synthesis and digestibility [35, 37]. Increasing levels of Alaria protein fraction decreased OMD [38], as supported by meta-analyses showing positive correlations between CH_4_ production and digestibility [39]. Supplemental OMD without additional VFA yield may reflect higher microbial protein synthesis or reduced hydrolysis of certain carbohydrates during *in vitro* incubation [38]. Brown macroalgae are generally less digestible than red macroalgae [40], due to complexes of sulfated polysaccharides, polyphenols, and proteins that are poorly degradable [41].

### Digestibility vs. methane mitigation trade-offs

A key finding is the divergence between species with high digestibility and those with strong methane-suppressing potential. Red macroalgae such as *E. spinosum*, *H. durvillei*, and *U. lactuca* exhibited the highest IVDMD and IVOMD, reflecting substantial microbial degradation of organic substrates and increased energy availability for the host. However, this enhanced degradation was often associated with higher methane production, particularly in *H. durvillei*. By contrast, *Sargassum* sp. showed lower digestibility but consistently reduced methane output. Species with moderate digestibility combined with robust antimethanogenic effects may therefore be more suitable as functional feed additives, while highly digestible species serve primarily as nutrient sources.

*H. durvillei* demonstrated some of the highest IVDMD and IVOMD values (Table 6), indicating that its organic matter was highly available to rumen microbes. This superior digestibility is likely attributable to its notably low crude fiber content (3.31%) and relatively high non-fiber extract proportion (25.79%), which facilitate rapid microbial breakdown and extensive fermentation.

### Chemical composition correlations and methane production

Nonetheless, the conditions that enhanced digestibility also facilitated methanogenesis. *H. durvillei* produced the maximum methane yield per unit of incubated dry matter and per unit of total gas, despite moderate overall gas production (Table 3). Its VFA profile showed a comparatively elevated acetate proportion and a high A/P ratio (A/P = 2.03), indicating excess metabolic hydrogen generation from fermentation pathways. This surplus hydrogen is subsequently utilized by methanogens, resulting in elevated methane production. Thus, the high methane output from *H. durvillei* is consistent with its superior digestibility: efficient substrate breakdown supplies both energy and hydrogen to rumen methanogens in the absence of inhibitory compounds that might restrict methanogenic activity.

**Figure 6:**
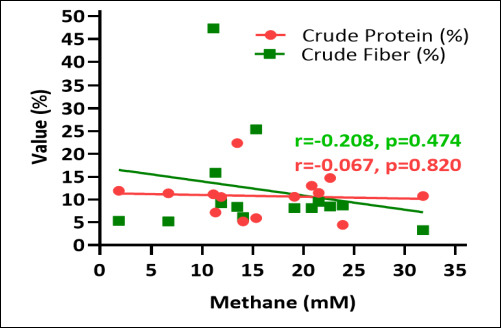
Correlation between estimated methane production (mM), crude protein (%), and crude fiber (%) of various macroalgae species.

The relationship between estimated methane production and macroalgal chemical composition is depicted in Figure 6. Estimated methane production exhibited a negative correlation with crude protein content and a positive correlation with crude fiber content. Macroalgae with elevated crude protein levels generally corresponded with reduced estimated methane production, whereas those with higher crude fiber content were associated with increased methane emissions.

**Figure 7:**
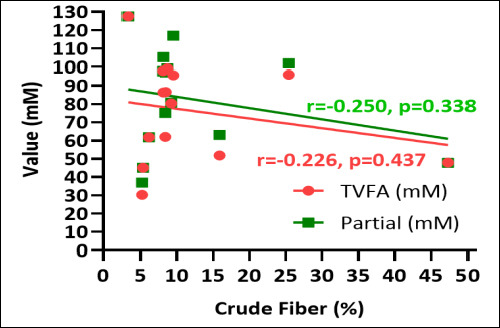
Correlation between total volatile fatty acid (TVFA) (mM), partial volatile fatty acid (short-chain fatty acid) (mM), and crude fiber (%) of various macroalgae species.

**Figure 8:**
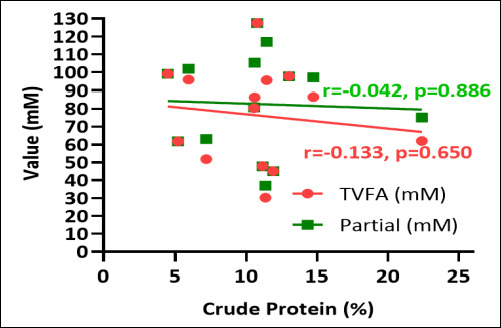
Correlation between total volatile fatty acid (TVFA) (mM), partial volatile fatty acid (short-chain fatty acid) (mM), and crude protein (%) of various macroalgae species.

Crude fiber content inversely correlated with both TVFA and individual SCFA concentrations. Elevated crude fiber levels were associated with decreased overall VFA production across the examined macroalgae species (Figure 7). Conversely, positive correlations were observed between crude protein content and TVFA and individual SCFA concentrations. Macroalgae with elevated crude protein levels correlated with enhanced TVFA production and increased individual SCFA concentrations. These findings demonstrate a consistent relationship between protein content and fermentation end-product generation (Figure 8).

### Factors contributing to interspecies variability

The substantial variability among macroalgae species can be attributed to several key factors. First, chemical composition differed widely, particularly in ash, crude fiber, and non-fiber extract contents, which directly influence rumen degradability and fermentation kinetics. High ash content, common in certain brown and red macroalgae, may dilute fermentable organic matter and limit microbial nutrient access. Second, cell wall structure and polysaccharide composition vary significantly among brown, green, and red macroalgae. Brown species are rich in alginates and fucoidans, red species contain agar and carrageenan, and green species are dominated by cellulose-based polysaccharides. These structural differences affect degradation rates and microbial colonization in the rumen [42]. Third, bioactive compounds, particularly in brown macroalgae such as *Sargassum* sp., likely contribute substantially to methanogenesis inhibition. Compounds including phlorotannins and halogenated metabolites have been shown to suppress methanogens or protozoa, thereby altering hydrogen utilization without fully inhibiting fermentation [43, 44].

### Phytochemical content and bioactive potential

Flavonoid content ranged from 0.08 to 1.50 mg quercetin/g, with *C. racemosa* (1.50 mg/g) and *Sargassum* sp. (1.42 mg/g) exhibiting the highest levels. Total phenolic content in methanol extracts ranged from 0.16 to 0.55 mg gallic acid equivalents (GA)/g. *U. lactuca* had the highest phenolic content (0.55 mg GA/g), followed by *H. durvillei* (0.44 mg GA/g) and *P. palmata* (0.39 mg GA/g). The elevated phenolic content in *U. lactuca* and *Halymenia* sp. may contribute to their high antioxidant capacity, consistent with the general richness of polyphenolic compounds in red and green macroalgae. Brown macroalgae, including *Sargassum* sp. and *Padina* sp., contained higher flavonoid levels than most red and green species, aligning with their known abundance of phlorotannins and other phenolic derivatives. The high flavonoid content in *Sargassum* sp. and *C. racemosa* positions these species as strong candidates for future bioactivity screening, given their prevalence in tropical oceans [45].

### Polyunsaturated fatty acids (PUFAs) and methane mitigation

The ability of macroalgal PUFAs to reduce enteric methane emissions during *in vitro* rumen fermentation is increasingly recognized. PUFAs suppress methanogenic archaea and reduce available hydrogen for methanogenesis through alternative pathways such as biohydrogenation. Significant PUFA levels are present in some macroalgae, particularly red species like *A. taxiformis*. Palmitic acid, a saturated fatty acid, can induce cell damage via lipotoxicity and apoptosis [46]. This contrasts with studies on *P. palmata*, which has high palmitic acid content (>13.2%). In addition, *P. palmata* exhibited the highest eicosapentaenoic acid (EPA) content, followed by *Alaria esculenta* and other species [47].

### Bioactive compounds and mechanisms of methane suppression

Although the fatty acid profile and phenolic content were not directly measured in the present study, the observed lower cumulative gas production and methane-related responses, particularly in the *Sargassum* sp. Treatment, may be partly explained by the presence of bioactive PUFAs and phenolic compounds commonly reported in this genus. These compounds suppress methanogenic archaea and reduce hydrogen availability during rumen fermentation, thereby modulating gas production kinetics.

PUFAs, abundant in certain brown macroalgae including *Sargassum* sp., inhibit methanogenesis by competing for hydrogen through pathways such as biohydrogenation, diverting it away from CH_4_ formation. Phenolic compounds (especially phlorotannins in brown macroalgae) exert direct antimicrobial effects on methanogens and protozoa, limiting hydrogen transfer to archaea while maintaining moderate fermentation. This selective action likely accounts for the characteristic profile of *Sargassum* sp.: moderate total gas combined with significantly reduced methane concentration, yield per gram DM, and methane-to-gas ratio.

These findings align with literature on brown macroalgae, where secondary metabolites drive enteric methane mitigation. The antimethanogenic effects in *Sargassum* sp. appear to stem primarily from microbial modulation and hydrogen sink redirection rather than broad suppression of substrate degradability. Direct measurement of PUFAs, phlorotannins, and other phenolics in future work would strengthen mechanistic understanding and support targeted use of Sargassum sp. as a functional feed additive for methane reduction in ruminant systems.

### Kinetics of gas production

No statistically significant differences in gas production were observed between 10 and 12 h of incubation (p = 0.067). These early periods likely reflect the latency phase of microbial fermentation, during which gas differences remain minimal. Gas production from *B. forbesii* and *G. gigas* was substantially higher than that from *T. ornata* and *H. durvillei*, which exhibited the lowest values. Fermentation rates in *B. forbesii* and *G. gigas* exceeded those in *T. ornata*. *B. forbesii*, *G. gigas*, and *Sargassum* sp. showed higher gas production, indicating greater potential for digestion and fermentation.

The highest asymptotic gas production (a + b) was recorded in *B. forbesii* (201.97 ± 32.92 mL), significantly exceeding values for most other macroalgae (p < 0.05). Gas production potential varied significantly among species (p < 0.05), as did the gas production rate constant (c) (p < 0.05; Table 7).

**Table 7:** *In vitro* rumen gas production of several macroalgae species (per incubation time, mL/g DM).

Macroalgae Species	Gas_10_ (mL/g DM)	Gas_12_ (mL/g DM)	Gas_24_ (mL/g DM)	Gas_36_ (mL/g DM)	Gas_48_ (mL/g DM)	Gas_72_ (mL/g DM)
Brown seaweeds						
*Padina* sp.	6.79 ± 3.96^ab^	6.79 ± 3.96^ab^	17.60 ± 5.94^ab^	28.39 ± 6.46^abc^	33.79 ± 6.02^ab^	41.39 ± 6.23^abc^
*Sargassum* sp.	11.20 ± 9.91^ab^	11.20 ± 9.91^ab^	33.59 ± 10.40^ab^	53.99 ± 11.60^fg^	62.99 ± 11.40^f^	77.79 ± 13.56^ef^
*Turbinaria ornata*	3.20 ± 3.83^a^	3.20 ± 3.83^a^	10.60 ± 5.85^a^	16.79 ± 6.61^a^	21.59 ± 6.80^a^	26.39 ± 7.89^a^
Green seaweeds						
*Boergesenia forbesii*	12.79 ± 5.45^b^	12.79 ± 5.45^b^	32.59 ± 8.79^b^	60.99 ± 10.08^g^	84.59 ± 7.58^g^	107.18 ± 9.85^g^
*Eucheuma cottonii*	7.79 ± 4.87^ab^	7.79 ± 4.87^ab^	20.19 ± 7.22^ab^	31.79 ± 7.32^abcd^	38.99 ± 8.18^abc^	46.59 ± 9.23^abc^
*Ulva lactuca*	9.99 ± 6.28^ab^	9.99 ± 6.28^ab^	30.99 ± 10.63^ab^	51.59 ± 9.63^efg^	60.38 ± 8.56^ef^	75.78 ± 9.40^ef^
Red seaweeds						
*Caulerpa racemosa*	9.60 ± 3.91^ab^	9.60 ± 3.91^ab^	22.40 ± 7.34^ab^	33.39 ± 7.13^abcde^	39.99 ± 8.25^bcd^	47.79 ± 9.20^bc^
*Eucheuma spinosum*	13.60 ± 5.77^b^	13.60 ± 5.77^b^	31.79 ± 8.04^b^	46.99 ± 10.95^cdefg^	53.78 ± 11.52^cdef^	61.78 ± 13.18^cde^
*Gelidium* sp.	8.39 ± 5.81^ab^	8.39 ± 5.81^ab^	19.39 ± 7.43^ab^	29.39 ± 8.53^abc^	35.79 ± 7.98^abc^	42.98 ± 9.77^abc^
*Gracilaria coronopifolia*	6.80 ± 9.31^ab^	6.80 ± 9.31^ab^	21.40 ± 14.64^ab^	36.60 ± 16.25^bcdef^	44.39 ± 16.90^bcde^	55.19 ± 17.78^cd^
*Gracilaria gigas*	11.79 ± 7.16^ab^	11.79 ± 7.16^ab^	38.19 ± 12.32^ab^	59.99 ± 12.31^g^	70.19 ± 11.48^fg^	83.18 ± 12.07^f^
*Gracilaria verrucosa*	10.59 ± 4.39^ab^	10.59 ± 4.39^ab^	29.80 ± 8.23^ab^	48.19 ± 8.44^defg^	57.79 ± 8.76^def^	70.59 ± 9.29^def^
*Halymenia durvillei*	6.79 ± 3.77^ab^	6.79 ± 3.77^ab^	15.39 ± 4.56^ab^	23.39 ± 4.67^ab^	28.79 ± 4.60^ab^	33.99 ± 4.69^ab^
*Palmaria palmata*	11.99 ± 8.18^ab^	11.99 ± 8.18^ab^	25.39 ± 11.73^ab^	35.99 ± 11.93^bcdef^	41.99 ± 12.38^bcd^	51.98 ± 14.62^bcd^
p-value	0.067	0.067	0.067	0.01	0.011	0.01

Means with different superscripts within a column are significantly different (p < 0.01 and p < 0.05). Values are presented as mean ± standard deviation. DM = Dry matter. Gas production values are expressed as mL per g of incubated dry matter (DM).

In this experiment, *Sargassum* sp., *U. lactuca*, and *G. gigas* displayed faster initial gas production rates, as evidenced by steeper early curves, but failed to surpass *B. forbesii* in overall cumulative production. Conversely, *T. ornata* exhibited the flattest curve, consistent with its low fermentability and reduced overall gas output. Cumulative gas production in *B. forbesii* was the highest, while *T. ornata* was the lowest, reflecting a clear pattern of limited fermentability in the latter. Significant differences in gas production emerged from 36 h onward, confirming distinct fermentability patterns among macroalgae species. These results demonstrate that *B. forbesii* ferments steadily, leading to a progressive increase in total gas volume over time.

**Figure 9:**
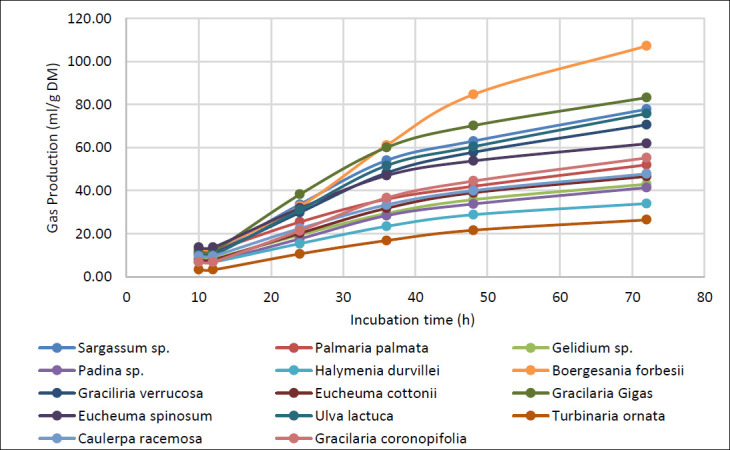
Cumulative gas production (mL/g DM) from the experimental macroalgae diets during the 72-h incubation period.

In contrast, *T. ornata* and *H. durvillei* consistently produced the lowest gas volumes, suggesting slower fermentation rates or restricted ruminal degradation. The total gas production curve (Figure 9) validates these observations. *B. forbesii* showed a linear and incremental increase in gas production throughout the 72-h incubation period, reaching maximum values at 72 h. The gas production parameters (Table 7) and cumulative curves (Figure 9) revealed significant differences in gas dynamics across macroalgae species.

*B. forbesii* likely contains more readily fermentable substrates accessible to rumen microorganisms, whereas *T. ornata* may possess more recalcitrant polysaccharides or inhibitory metabolites that limit fermentation. Higher gas production rates were observed in *G. gigas*, *Sargassum* sp., *U. lactuca*, and *C. racemosa*, indicating more intense substrate fermentation in these species. Notably, *B. forbesii* had the lowest rate constant (c = 0.01 mL/h) despite its superior cumulative production, suggesting slower but more sustained fermentation.

High-yield but slower-fermenting species like *B. forbesii* could provide sustained energy release in ruminant diets, while low-yield species such as *T. ornata* may limit total fermentation. These variations in fermentation kinetics and gas volume could be strategically exploited to modulate rapid fermentation, reduce digestive disturbances, and balance energy supply in ruminant rations.

The asymptote (a + b), rate constant (c), and cumulative gas production decreased as macroalgae inclusion level increased in the substrate, consistent with literature showing general declines in cumulative gas production (CGP) and fermentation with higher inclusion rates. Kinley *et al*. [48] reported reduced *in vitro* gas production with *Asparagopsis* inclusion, with asymptotic values of 18 mL/g OM for *L. digitata* and 38 mL/g OM for *P. palmata* [49]. Maximal gas production in the present study was approximately 10-fold higher, likely due to methodological differences, although inter- and intraspecies variation in chemical composition is expected to influence gas output. The primary driver of potential total gas production (a + b) is carbohydrate fermentation into VFAs (acetate, propionate, butyrate). This may be enhanced by phytonutrients in certain substrates, including phenolics, flavonoids, and antioxidants, which interact positively with fiber and protein to promote ruminal fermentation and gas production [50].

Macroalgae species significantly influenced both total gas produced and production rate (p < 0.05 for a + b and c). *B. forbesii* and *G. gigas* consistently showed the highest cumulative gas production from 36 to 72 h, indicating sustained microbial activity. In contrast, *T. ornata* exhibited the lowest rate throughout incubation, reflecting poor degradability and restricted fermentation (Table 8).

**Table 8:** Gas production parameters derived from varied macroalgae diets over 72 h of incubation.

Macroalgae Species	a + b (mL)	c (mL/h)
Brown seaweeds		
*Padina* sp.	55.35 ± 8.56^abc^	0.02 ± 0.008^abc^
*Sargassum* sp.	97.37 ± 23.14^ef^	0.03 ± 0.007^bc^
*Turbinaria ornata*	36.12 ± 10.58^a^	0.02 ± 0.009^abc^
Green seaweeds		
*Boergesenia forbesii*	201.97 ± 32.92^g^	0.01 ± 0.002^a^
*Caulerpa racemosa*	59.99 ± 8.68^abc^	0.03 ± 0.004^bc^
*Ulva lactuca*	100.30 ± 20.31^f^	0.03 ± 0.009^bc^
Red seaweeds		
*Eucheuma cottonii*	58.39 ± 9.47^abc^	0.02 ± 0.002^ab^
*Eucheuma spinosum*	69.65 ± 14.95^bcd^	0.03 ± 0.002^bc^
*Gelidium* sp.	55.14 ± 11.31^abc^	0.02 ± 0.006^ab^
*Gracilaria coronopifolia*	67.77 ± 6.68^cde^	0.02 ± 0.000^ab^
*Gracilaria gigas*	97.04 ± 9.39^ef^	0.03 ± 0.006^c^
*Gracilaria verrucosa*	90.02 ± 14.82^def^	0.02 ± 0.01^bc^
*Halymenia durvillei*	44.29 ± 8.83^ab^	0.02 ± 0.008^abc^
*Palmaria palmata*	56.98 ± 9.95^bc^	0.02 ± 0.005^abc^
p-value	0.011	0.01

Means with different superscripts within a column are significantly different (p < 0.01 and p < 0.05). Values are presented as mean ± standard deviation. a + b = Asymptotic (potential) gas production (mL); c = Gas production rate constant (mL/h).

### Limitations and practical implications

Despite consistent trends in gas production kinetics, this study was conducted under *in vitro* conditions, which may not fully capture *in vivo* rumen dynamics, including feed intake regulation, digesta passage, and host–microbiome interactions. Bioactive compounds potentially involved in methane suppression (e.g., specific fatty acids and phenolics) were not quantified directly.

Macroalgae should not be considered a uniform ruminant feed resource. Highly digestible species such as *E. spinosum*, *U. lactuca*, and *H. durvillei* offer strong potential as alternative nutrient sources in tropical systems with seasonal forage limitations. However, their tendency to increase methane emissions requires careful control of inclusion levels. *Sargassum* sp. appears better suited as a functional additive rather than a primary ingredient, given its robust methane-reducing capacity despite moderate digestibility. Strategic low-level inclusion of such species could reduce enteric methane while preserving rumen function and animal productivity.

The positive correlation between crude protein content and TVFA concentration/ammonia production aligns with prior research on protein-rich macroalgae and unconventional ruminant feeds [51]. Conversely, the inverse relationship between crude fiber content and total gas production, VFA concentration, and digestibility is consistent with the inhibitory effects of structural carbohydrates on rumen fermentation, as observed in other macroalgae studies. If it is compared to other roughage or forage that the most significant gas production of hay was observed during the 21-day harvesting interval, as well as IVDMD, which contributed to a higher potential for degradation. This occurred as a result of the substantial quantity of soluble material, as the soluble fractions in feed are responsible for the production of volatile fatty acids, which are the primary source of energy for ruminants. The converse condition was observed during the extended cutting intervals; this can be attributed to the degradability of the NDF level, ADF, and lignin contents [52].

 A previous study [53], which was about cassava tops (CT) and cassava pulp (CS), there were some nutrient parameters that were different, which may be attributed to the effects of certain compounds in CT and CS on the fermentation process or the varying levels of additive source supplementation. However, those nutrient contents can be utilized as animal feedstuffs to enhance animal productivity.

 The gas parameters in the equation account for 74% of the variability in the dry matter effective degradability of the forages, as indicated by the coefficient of determination of 0.740 in the predictor model for the Dry Matter Effective degradability. There was a reduced coefficient of determination for the predictor model for organic matter effective degradability, which was 0.659. The coefficient of determination for effective degradability of CP also showed correlations with cumulative gas productions was the highest, at 0.813. This is a rationale for the conclusion that the majority of the gas generated during fermentation in the rumen is a result of the rumen microbes’ degradation of plant cell wall components. Conversely, the coefficient of determination for the CP ED predictor was only 0.500 [54].

These results suggest that targeted integration of highly digestible and methane-suppressing macroalgae species could provide a balanced strategy for improving feed efficiency and reducing environmental impact in tropical ruminant systems. Further *in vivo* studies are essential to validate optimal inclusion rates, animal performance, long-term safety, methane emissions, nutrient utilization, and practical feeding outcomes.

## CONCLUSION

This study provides the first comprehensive *in vitro* evaluation of 14 native Indonesian macroalgae species as potential ruminant feed resources, focusing on rumen fermentation profiles, digestibility, gas production kinetics, and enteric methane mitigation. The results reveal significant species-specific variations that underscore the diverse potential of macroalgae in sustainable livestock nutrition.

*Boergesenia forbesii* exhibited the highest total gas production (201.97 ± 32.92 mL) and fermentability, indicating superior degradation by rumen microorganisms, while *Turbinaria ornata* showed the lowest gas output, likely due to high fiber or mineral content limiting microbial access. Highly digestible species, such as *Halymenia durvillei* and *Eucheuma spinosum*, achieved the greatest IVDMD and IVOMD (p < 0.01), providing enhanced nutrient availability, but they also produced elevated methane levels (up to 94.11% higher than low-methane species). In contrast, *Sargassum* sp. and *Caulerpa racemosa* emerged as top candidates for methane mitigation, with *Sargassum* sp. recording the lowest methane concentration, yield per gram DM, and methane-to-gas ratio (reductions of 71.86–94.11%), despite moderate fermentability. VFA profiles varied markedly: *H. durvillei* yielded the highest total VFA (127.51 mM) with acetate-dominant patterns favoring methanogenesis, while *B. forbesii* and *H. durvillei* promoted propionate (an alternative hydrogen sink). Correlations confirmed negative associations between methane and propionate/crude protein content, and positive links with acetate/crude fiber. PF and microbial protein synthesis were highest in *B. forbesii* and *E. spinosum*, respectively, reflecting efficient nutrient partitioning. Phytochemicals like phenolics (highest in *U. lactuca* at 0.55 mg GA/g) and flavonoids (highest in *C. racemosa* at 1.50 mg quercetin/g) likely contributed to antimethanogenic effects in brown species.

These findings highlight macroalgae as viable, sustainable alternatives to conventional forages in tropical ruminant systems, where forage scarcity is common. High-digestibility species (*H. durvillei*, *E. spinosum*, *U. lactuca*) could serve as nutrient-rich supplements to improve energy and protein supply, enhancing animal productivity. Methane-suppressing species (*Sargassum* sp. and *C. racemosa*) offer practical value as functional additives for reducing enteric CH_4_ emissions, a critical step toward lowering livestock’s greenhouse gas footprint without compromising fermentation. Strategic blending (e.g., 1–5% inclusion of *Sargassum* sp. with digestible reds/greens) could balance nutrition, methane mitigation, and cost-effectiveness, particularly in Indonesia’s coastal regions with abundant macroalgae resources. This approach supports eco-friendly farming, potentially reducing reliance on imported feeds and promoting local biodiversity utilization.

The study’s robustness lies in its comparative design across a diverse set of 14 native species, integrating multiple parameters (fermentation, digestibility, kinetics, methane) using standardized *in vitro* methods. This provides a foundational dataset for Indonesian macroalgae, previously underexplored, and incorporates correlations with chemical composition for mechanistic insights. The use of advanced statistical analysis (e.g., p < 0.01 significance) and visualizations (Figures 3–9) enhances interpretability, offering a model for similar evaluations in other regions.

As an *in vitro* study, results may not fully replicate *in vivo* conditions, including digesta passage, host-microbiome interactions, and long-term effects on animal health/productivity. Bioactive compounds (e.g., PUFAs, phenolics) were inferred but not directly quantified, limiting precise mechanistic attribution. The focus on Indonesian species restricts generalizability, and high ash/fiber in some browns may overestimate inhibition in mixed diets.

*In vivo* trials are essential to validate methane reductions, optimal inclusion rates (e.g., 1–10% DM), and impacts on animal performance, milk/meat quality, and gut health. Further research should quantify bioactives (phlorotannins, PUFAs) and microbial shifts using metagenomics, explore processing techniques (drying, grinding) to enhance digestibility, and assess economic viability for smallholder farmers. Long-term studies on environmental sustainability (e.g., macroalgae harvesting impacts) and multi-species blends could optimize formulations for methane abatement while maintaining productivity.

In conclusion, native Indonesian macroalgae represent a promising, underutilized resource for ruminant nutrition, with species like *Sargassum* sp. offering effective methane mitigation and others like *H. durvillei* providing superior digestibility. By leveraging these natural feed additives, tropical livestock systems can advance toward greater sustainability, reducing environmental footprints while supporting food security. This research paves the way for innovative, bio-based strategies in animal agriculture, aligning with global efforts to combat climate change through eco-friendly feed solutions.

## DATA AVAILABILITY

The data supporting the findings of this study are available upon reasonable request from the corresponding author.

## AUTHORS’ CONTRIBUTIONS

DSW: Investigation, validation, data curation, formal analysis, and writing of the original draft. KGW: Conceptualization, supervision, and writing of the review and editing. RR: Conceptualization, supervision, and writing and review of the manuscript. GG: Validation, data analysis, and writing of the manuscript and editing. APS: Validation and writing of the manuscript and editing. MS: Validation and writing of the manuscript and editing. RF and AF: Executed the experiment, performed laboratory analysis, and analyzed the data. DP: Validation and writing, review, and editing of the manuscript. WP: Data analysis and writing, review, and editing of the manuscript. GKA: Experiment execution, laboratory analysis, data analysis, and editing. YW: Validation and writing, review, and editing of the manuscript. AJ: Conceptualization, supervision, methodology, and writing, review, and editing of the manuscript. All authors have read and approved the final version of the manuscript.
